# Tbx1 represses *Mef2c* gene expression and is correlated with histone 3 deacetylation of the anterior heart field enhancer

**DOI:** 10.1242/dmm.029967

**Published:** 2018-08-30

**Authors:** Luna Simona Pane, Filomena Gabriella Fulcoli, Andrea Cirino, Alessandra Altomonte, Rosa Ferrentino, Marchesa Bilio, Antonio Baldini

**Affiliations:** 1 CNR Institute of Genetics and Biophysics Adriano Buzzati Traverso, Via Pietro Castellino 111, 80131 Napoli, Italy; 2 Department of Molecular Medicine and Medical Biotechnology, University of Naples Federico II, 80131 Napoli, Italy

**Keywords:** Anterior heart field enhancer, Mef2c, Tbx1

## Abstract

The *TBX1* gene is haploinsufficient in 22q11.2 deletion syndrome (22q11.2DS), and genetic evidence from human patients and mouse models points to a major role of this gene in the pathogenesis of this syndrome. Tbx1 can activate and repress transcription, and previous work has shown that one of its functions is to negatively modulate cardiomyocyte differentiation. Tbx1 occupies the anterior heart field (AHF) enhancer of the *Mef2c* gene, which encodes a key cardiac differentiation transcription factor. Here, we show that increased dosage of *Tbx1* correlates with downregulation of *Mef2c* expression and reduced acetylation of its AHF enhancer in cultured mouse myoblasts. Consistently, 22q11.2DS-derived and *in vitro*-differentiated human induced pluripotent stem cells (hiPSCs) expressed higher levels of *MEF2C* and showed increased AHF acetylation, compared with hiPSCs from a healthy donor. Most importantly, we show that in mouse embryos, loss of *Tbx1* enhances the expression of the Mef2c-AHF-Cre transgene in a specific region of the splanchnic mesoderm, and in a dosage-dependent manner, providing an *in vivo* correlate of our cell culture data. These results indicate that Tbx1 regulates the Mef2c AHF enhancer by inducing histone deacetylation.

## Abstract (precis)

**Summary:** Using three experimental models, we demonstrate that Tbx1 suppresses *Mef2c* gene expression, and show that histone 3 acetylation of the anterior heart field enhancer of the *Mef2c* gene responds negatively to Tbx1 dosage.

## INTRODUCTION

During development, cardiac progenitors of the second heart field (SHF) are recruited or incorporated into the cardiac outflow tract and right ventricle (Kelly et al., 2001; Mjaatvedt et al., 2001; Waldo et al., 2001). During this process, cells activate a differentiation program that leads to expression of specialized proteins, such as contractile proteins necessary for cardiomyocyte function. This process, still to be dissected in detail, should be tightly regulated so that a sufficient number of progenitors are allowed to proliferate and are prevented from differentiating prematurely. These two basic functions (pro-proliferative and anti-differentiative) are likely effected by a combination of signals and transcription factors, among which Tbx1 is a major candidate (Jerome and Papaioannou, 2001; Lindsay et al., 2001; Merscher et al., 2001; Rana et al., 2014). Indeed, Tbx1 is expressed in cardiac progenitors of the SHF, is shut down as progenitors differentiate, and positively regulates pro-proliferative signals and negatively regulates pro-differentiative factors, such as *Gata4* (Liao et al., 2008; Pane et al., 2012), *Mef2c* (Pane et al., 2012), Srf (Chen et al., 2009) and Smad1 (Fulcoli et al., 2009). Downregulation of Srf and Smad1 signaling is effected through nontranscriptional mechanisms, but *Mef2c* gene expression appears to be transcriptionally repressed by Tbx1.

Tbx1 regulates its target genes by interacting with the SWI-SNF-like BAF complex and with histone methyltransferases (Stoller et al., 2010; Chen et al., 2012; Fulcoli et al., 2016; Baldini et al., 2017). However, there are a number of questions to be answered. In this work, we addressed the question as to how Tbx1 represses *Mef2c* expression. We used three different model systems: (1) a mouse myoblast cell line, (2) transgenic mouse embryos and (3) differentiating human induced pluripotent stem cells (hiPSCs) from a 22q11.2 deletion syndrome (22q11.2DS) patient and a healthy donor. Results indicate that transcriptional repression is associated with reduced histone 3 acetylation in the region bound by Tbx1 in cultured cells, which is the previously defined anterior heart field (AHF) enhancer of the *Mef2c* gene (Dodou et al., 2004). In addition, we show that, *in vivo*, Tbx1 regulation of the AHF enhancer is dosage dependent and regionally restricted, suggesting that there are crucial interactions with other transcription factors in regulating this enhancer.

## RESULTS

### Increased Tbx1 expression is associated with reduced histone 3 acetylation of the *Mef2c* AHF enhancer sequence

**Fig. 1.**
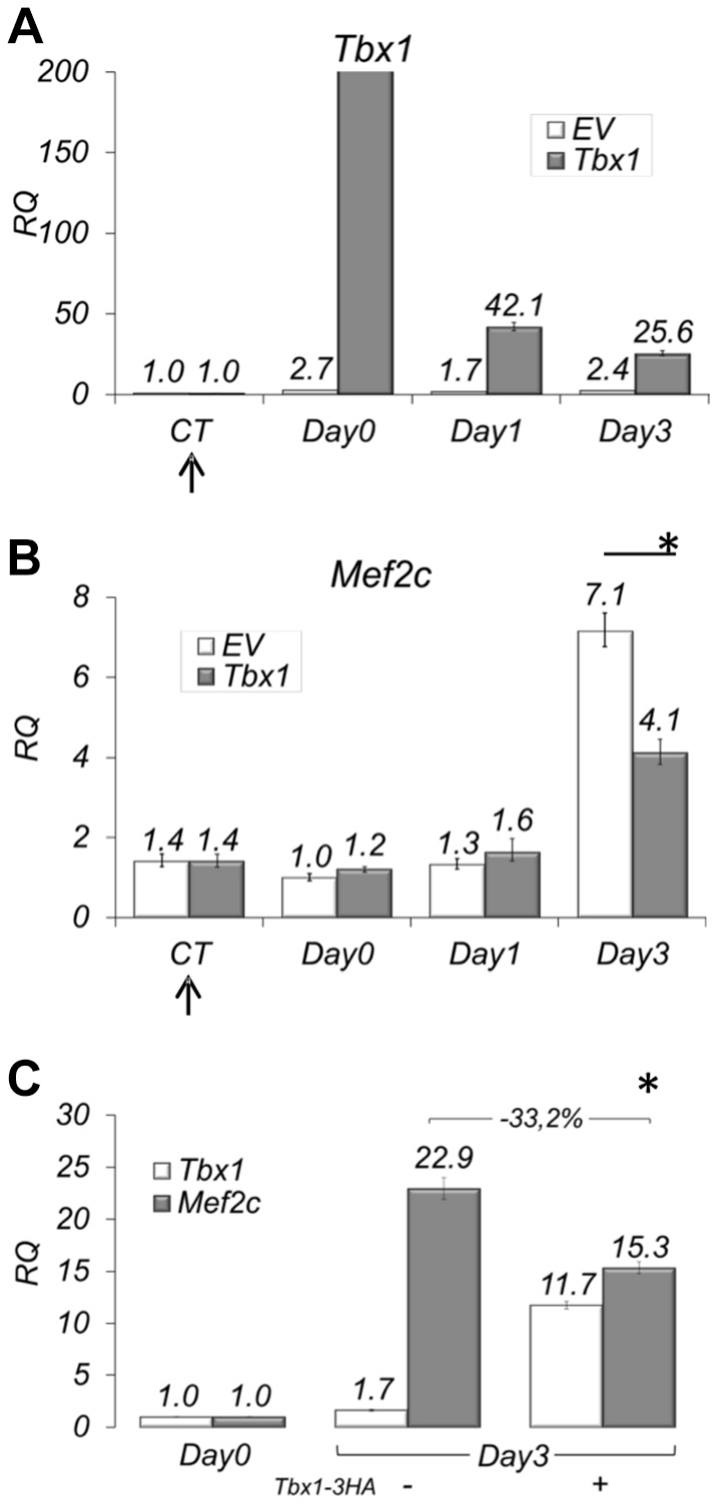
**Tbx1 negatively regulates** ***Mef2c* expression in C2C12 at day 3.** (A,B) Time course of *Tbx1* (A) and *Mef2c* (B) expression during *in vitro* C2C12 myoblast differentiation, with or without transient transfection of *Tbx1*. Note that Tbx1 negatively regulates *Mef2c* expression at day 3 of differentiation (B). (C) *Mef2c* mRNA levels are affected by *Tbx1* overexpression 24 h after transfection (performed at day 2). Data are from three experiments (mean±s.d.); *P<0.05. The arrows in A and B indicate the time of transfection (24 h before induction of differentiation, which is on day 0).

To understand the mechanisms by which Tbx1 represses *Mef2c*, we used mouse myoblast C2C12 cells undergoing muscle differentiation. We transfected a Tbx1 expression vector in these cells at day 0 and let them differentiate (Fig. 1A). The *Mef2c* gene was strongly upregulated at day 3 of differentiation in control cells, but in *Tbx1*-transfected cells this upregulation was strongly reduced (Fig. 1B). To exclude that this effect might be due to a delayed differentiation caused by early *Tbx1* transfection, we repeated the experiment by transfecting Tbx1 after the initiation of differentiation, at day 2, and assayed *Mef2c* expression at day 3. Also in this case, we observed a strong reduction of *Mef2c* expression (Fig. 1C).

**Fig. 2.**
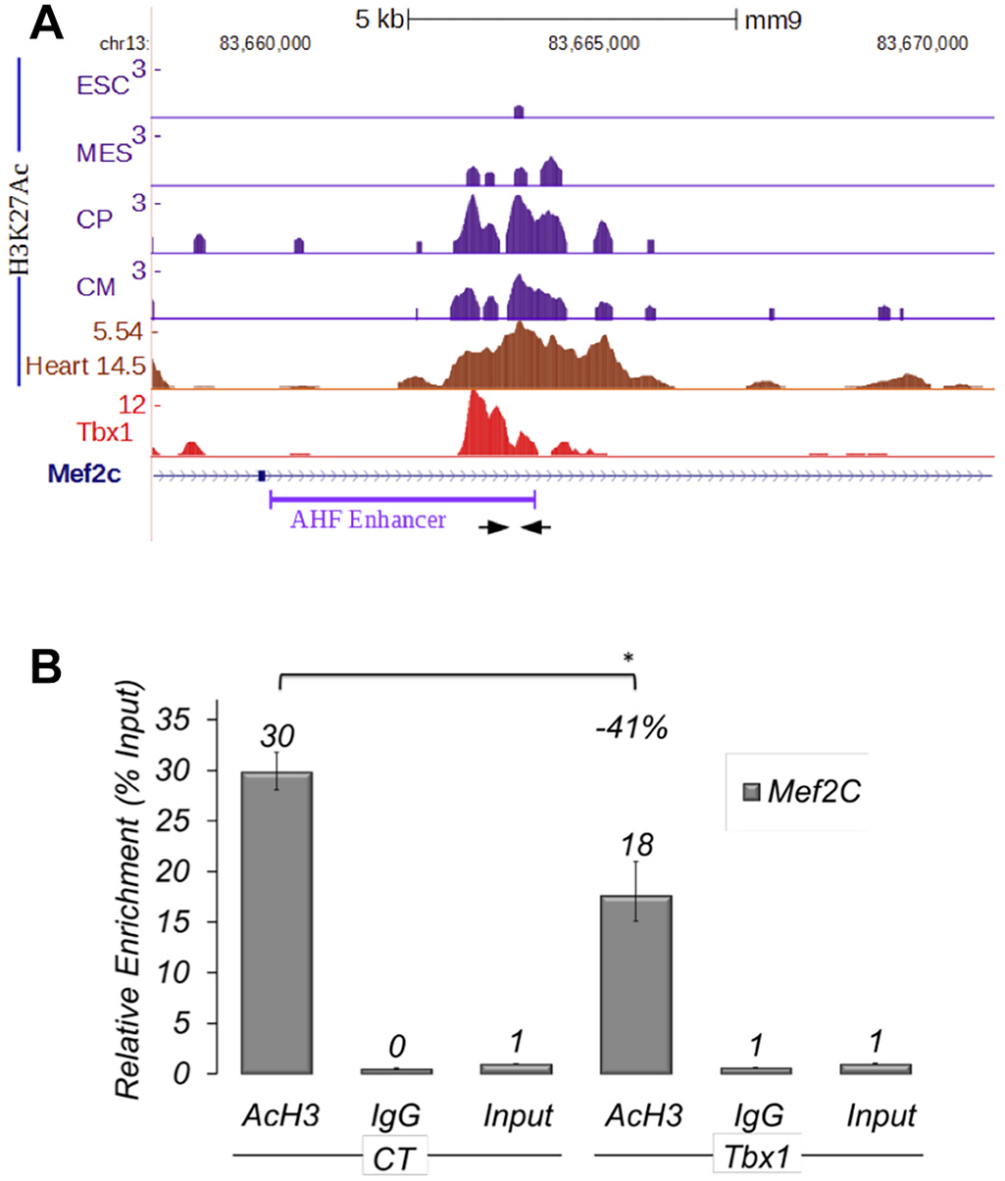
**Acetylation of histone 3 in the AHF enhancer region of *Mef2c* is reduced by *Tbx1* overexpression.** (A) Examples of ChIP-seq data profiles as shown using the UCSC genome browser (https://genome.ucsc.edu) in the genomic region containing the AHF enhancer of *Mef2c* (GenBank AY324098): H3K27Ac profiles at four stages of cardiomyocyte differentiation of mouse ESCs: undifferentiated ESCs (ESC), cells expressing mesodermal markers (MES), cardiac precursors (CP), cardiomyocytes (CM); H3K27Ac ChIP-seq data profiles in E14.5 heart tissue; Tbx1 ChIP-seq data profiles. Arrows indicate the primers used for real-time PCR amplification. (B) q-ChIP assay of AcH3 on differentiating C2C12 cells (day 3) transfected with an empty vector (EV) or with a vector overexpressing Tbx1 (Tbx1), followed by quantitative real-time PCR. Data are from three experiments (mean±s.d.); *P<0.05.

Tbx1 chromatin immunoprecipitation (ChIP) with DNA sequencing (ChIP-seq) data obtained on P19Cl6 cells (Fulcoli et al., 2016) and previously published quantitative ChIP (q-ChIP) data on C2C12 cells (Pane et al., 2012) revealed enrichment at the *Mef2c* AHF enhancer, as previously defined (Dodou et al., 2004) (Fig. 2A). In particular, by comparing Tbx1 ChIP-seq data on P19Cl6 cells with histone modification data during cardiac differentiation of mouse embryonic stem cells (ESCs) (Wamstad et al., 2012), we noted that the Tbx1-enriched region has relatively low H3K27Ac expression in ESCs but progressively becomes H3K27Ac-rich during cardiac differentiation. Using available data from ENCODE, we also noted that the same region is acetylated at embryonic day (E) 14.5 in the heart, where *Tbx1* is not expressed (Fig. 2A). These observations suggest that, in this region, H3 acetylation increases as differentiation progresses. Thus, we reasoned that Tbx1, directly or indirectly, might negatively regulate acetylation in this region. To test this possibility, we performed q-ChIP with an antibody against H3 acetylation (anti-H3-Ac) in C2C12 cells. Results showed that *Tbx1* overexpression substantially reduced H3-Ac enrichment at the *Mef2c* AHF enhancer (Fig. 2B). Consistent results were obtained using antibodies against H3K27Ac on C2C12 cells after *Tbx1* knockdown, which increased H3K27Ac enrichment (Fig. S1). We addressed the question as to whether the H3 deacetylating effect of Tbx1 might be due to a direct interaction with HDAC1 or HDAC2. However, repeated co-immunoprecipitation (IP) experiments failed to demonstrate HDAC1-Tbx1 or HDAC2-Tbx1 co-IP, suggesting that deacetylation is an indirect effect or that Tbx1 interacts with other HDACs (Fig. S2).

These results suggest that Tbx1 contrasts *Mef2c* AHF enhancer activity by negatively regulating its H3 acetylation.

### Loss of Tbx1 is associated with expansion of transgenic Mef2c-AHF-Cre expression *in vivo*

**Fig. 3.**
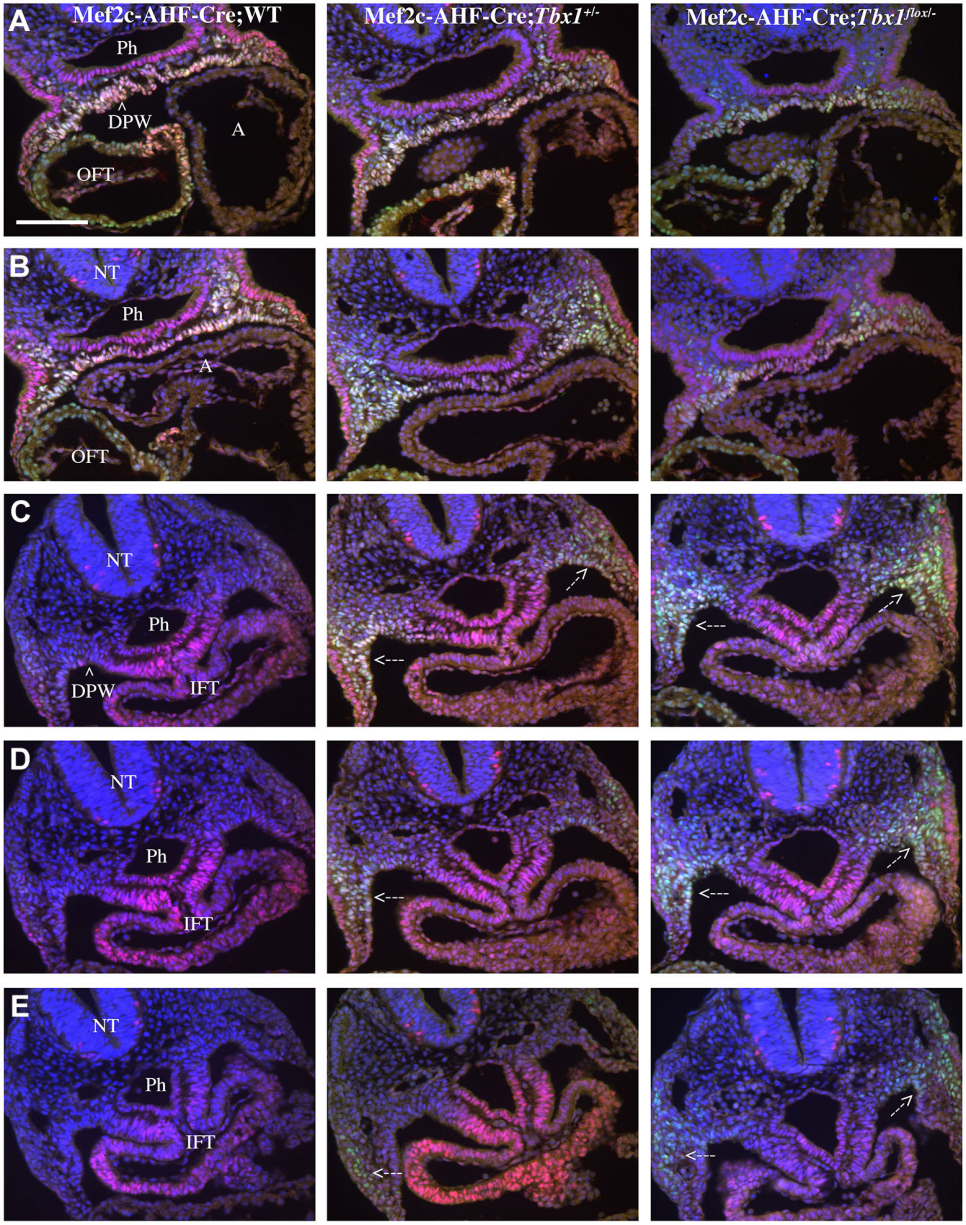
**Tbx1 regulates the Mef2c AHF enhancer *in vivo*.** (A-E) Immunofluorescence of transverse sections of E9.5 (20 somites) embryos with the genotype indicated, using anti-Cre (green) and anti-Isl1 (red) antibodies. The first row of sections (A) corresponds to a level immediately below the outflow tract. The second row (B) is just anteriorly to the inflow tract, while the third fourth and fifth rows (C-E) correspond to the inflow tract. Note that the Cre signals in A and B are comparable in the three genotypes, while in C-E, reduced dosage of *Tbx1* is associated with expansion of Cre expression (dashed line arrows). We examined three embryos per genotype. Fig. S3 shows *Tbx1* gene expression in similar sections. A, atrium; DPW, dorsal pericardial wall (line arrowhead); IFT, inflow tract; NT, neural tube; OFT, outflow tract; Ph, pharynx. Scale bar: 100 μm.

**Fig. 4.**
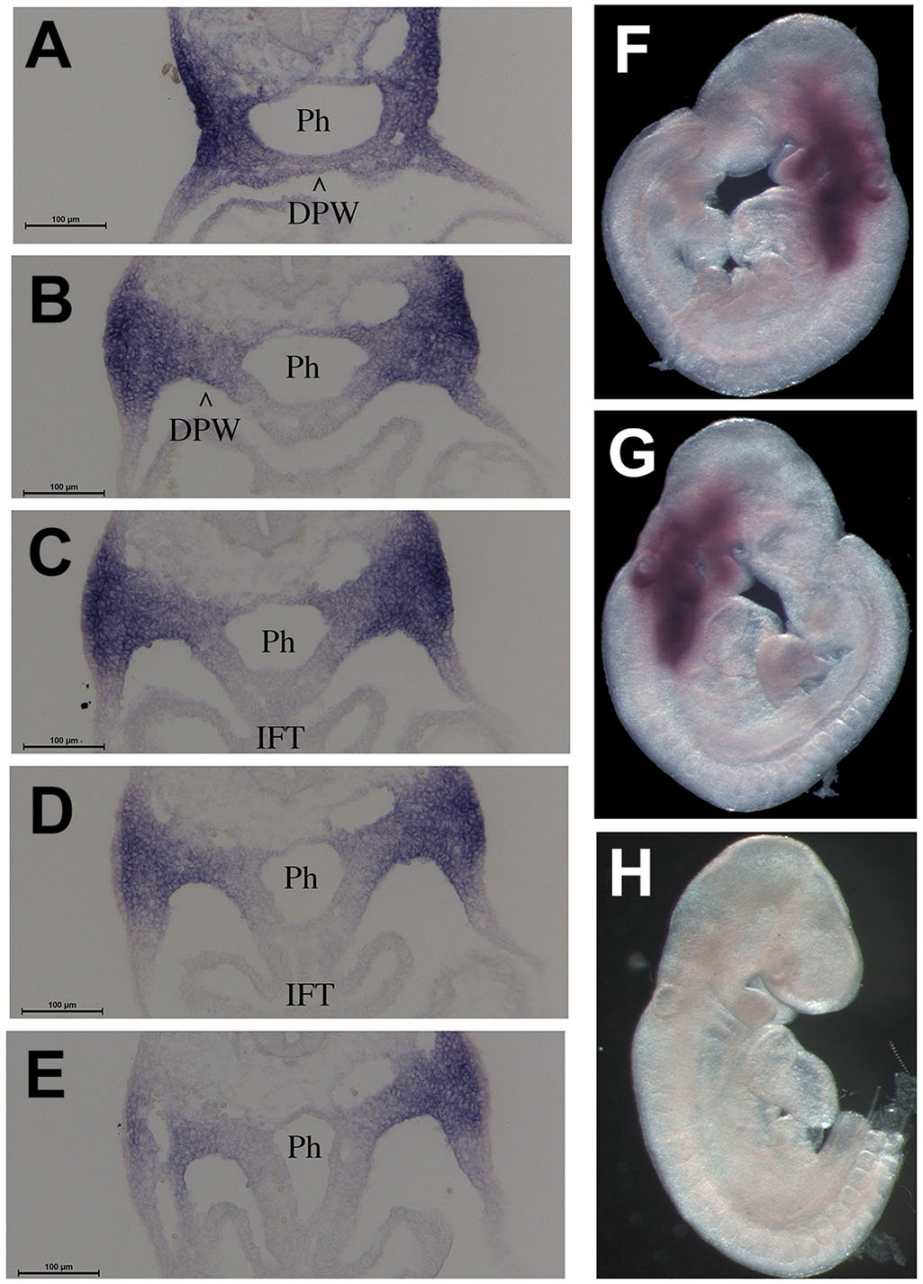
**Transverse sections of a *Tbx1*^+/lacZ^ embryo at E9.5 (22 somites) after staining with the β-gal chromogenic substrate salmon-gal.** (A-E) Sections are at similar levels as in Fig. 3A-E. Scale bars: 100 µm. (F,G) Left and right sides of the whole-mount stained embryo from which sections in A-E were derived. (H) Wild-type littermate with negative staining. DPW, dorsal pericardial wall; IFT, inflow tract; Ph, pharynx.

To determine whether Tbx1 represses the activity of the Mef2c AHF enhancer *in vivo*, we used the transgenic Mef2c-AHF-Cre line (Verzi et al., 2005), which carries the AHF enhancer that drives the expression of Cre recombinase. The activity of the enhancer was detected using immunofluorescence with an anti-Cre antibody on embryo sections. We tested Mef2c-AHF-Cre;*Tbx1*^+/+^ (control), Mef2c-AHF-Cre;*Tbx1*^+/−^ (heterozygous) and Mef2c-AHF-Cre;*Tbx1^flox^*^/−^ embryos (homozygous null in the Mef2c-AHF-Cre expression domain, and heterozygous elsewhere). We found that at E9.5 (20 somites), Mef2c-AHF-Cre was expressed in the core mesoderm of the first and second pharyngeal arches (PAs), in the cardiac outflow tract (OFT), in the SHF (mostly anterior) and in a posterior-lateral cell population of the splanchnic mesoderm at the level of the cardiac inflow tract inlet, in continuity with the SHF (Fig. 3). Cre expression in the more anterior domains (PAs, OFT and SHF) was comparable between control and mutant embryos (Fig. 3). However, more posteriorly, Cre expression was expanded in both heterozygous and homozygous mutants, albeit more evident in the latter (Fig. 3). The region of expanded Mef2c-AHF-Cre expression is well within the *Tbx1* expression domain at this stage (Fig. 4). Thus it is possible that Tbx1 regulates the enhancer directly.

### Increased expression of *MEF2C* in 22q11.2DS-derived hiPSCs upon cardiac differentiation

**Fig. 5.**
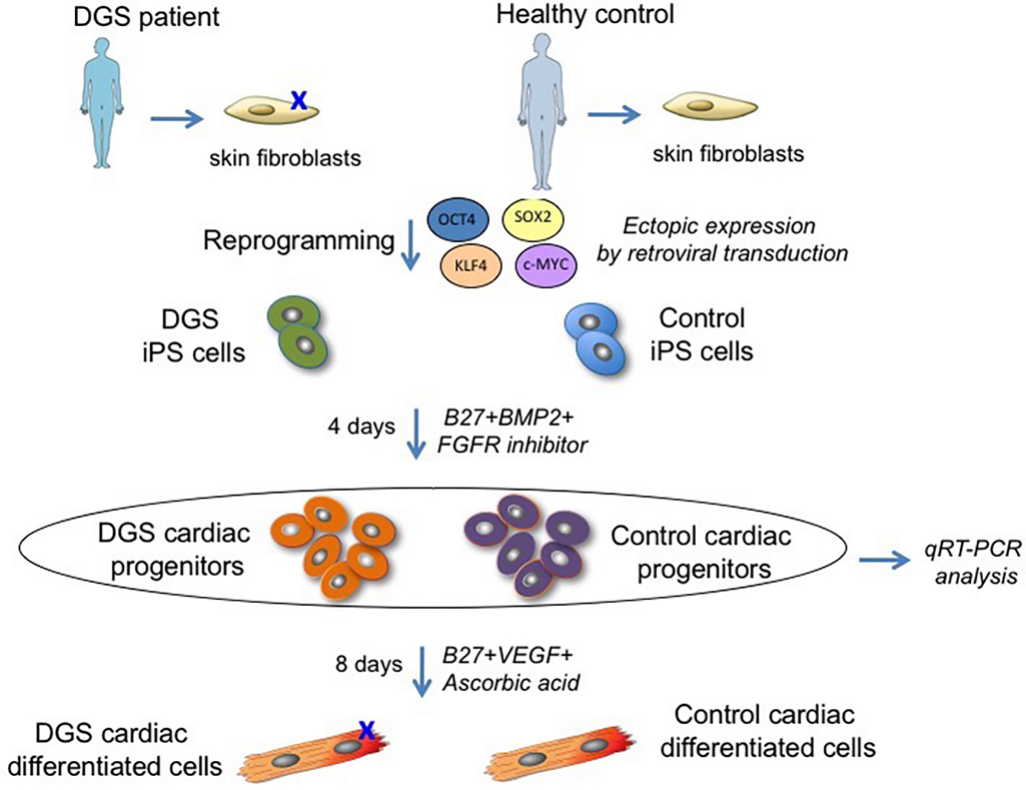
**Experimental design for the generation and cardiac differentiation of control and 22q11.2DS hiPSCs.** hiPSCs were generated by overexpression of retroviral transgenes for *OCT3/4*, *SOX2* and *KLF4* in primary skin fibroblasts from a 22q11.2DS (DGS) patient and from an unrelated healthy donor. Two hiPSCs clones from each individual were differentiated in cardiac progenitors and analyzed by qRT-PCR.

To determine whether the above observations are also relevant for human cells, we have generated induced pluripotent stem cells (hiPSCs) from a 22q11.2DS patient and from an unrelated healthy donor (Fig. 5). hiPSC lines were characterized as shown in Figs S3 and S4. Two hiPSC clones were differentiated into cardiac progenitors using a previously described protocol (Leschik et al., 2008; Moretti et al., 2010). We measured *TBX1* expression and found that it peaked on day 4 of differentiation (Fig. 7A), when cells also expressed other markers of cardiac progenitors (Fig. S5b). We observed that 22q11.2DS-derived cell lines expressed a lower level of *TBX1*, as the deletion removes one copy of the gene (Fig. S5A).

**Fig. 6.**
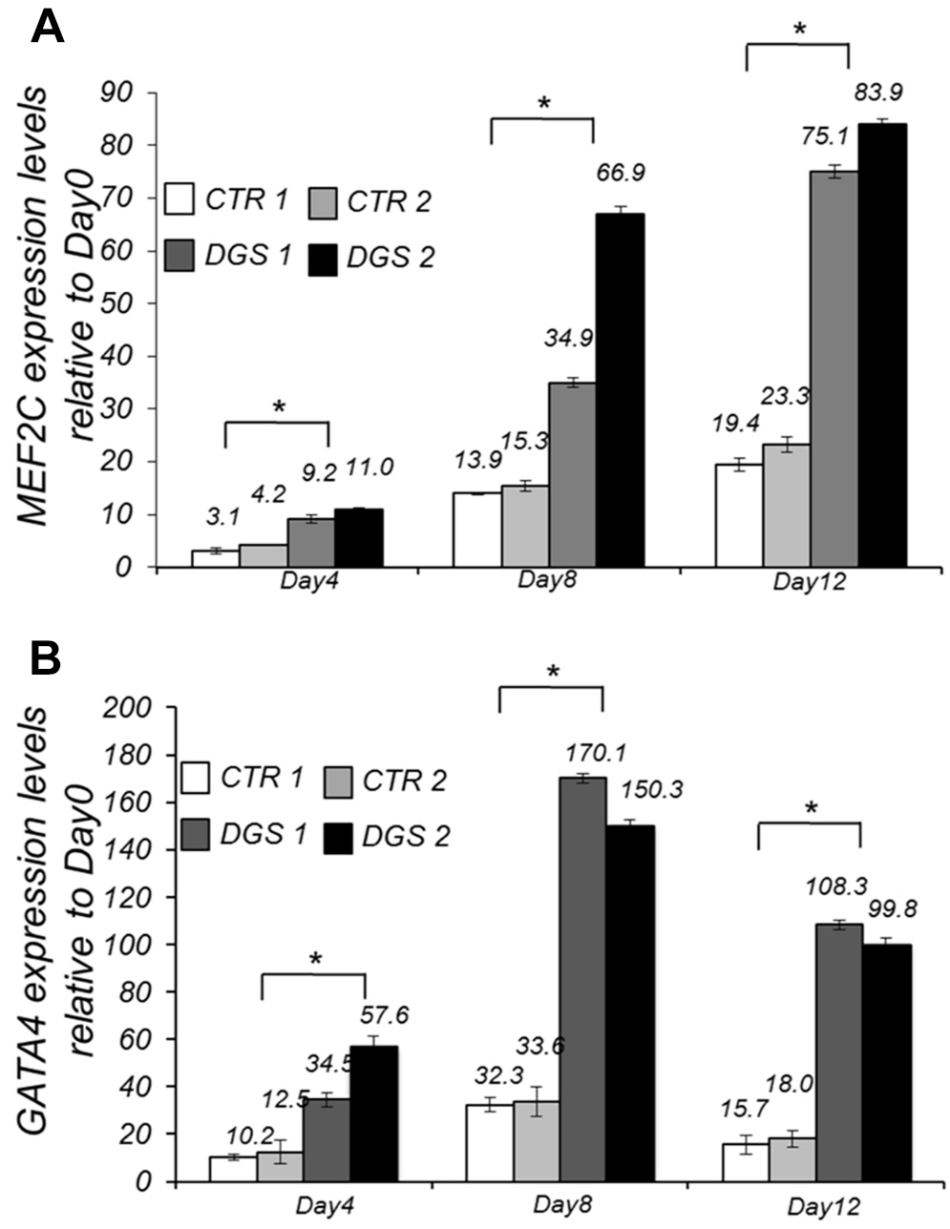
**Analysis of *MEF2C* and *GATA4* expression in control and 22q11.2DS hiPSC-derived cardiac cells.** (A,B) qRT-PCR-based *MEF2C* (A) and *GATA4* (B) expression in two hiPSC clones from a control (CTR) individual and two from a 22q11.2DS (DGS) patient at days 4, 8 and 12 of cardiac differentiation. *n*=3, *P<0.05 vs day 0.

We measured the expression of *MEF2C* and *GATA4*, which is another target of Tbx1 (Liao et al., 2008; Pane et al., 2012), by quantitative reverse transcription PCR (qRT-PCR) on days 4, 8 and 12 of differentiation, and found that both genes are upregulated in patient-derived cells at all differentiation points tested, compared with control cells (Fig. 6A,B).

**Fig. 7.**
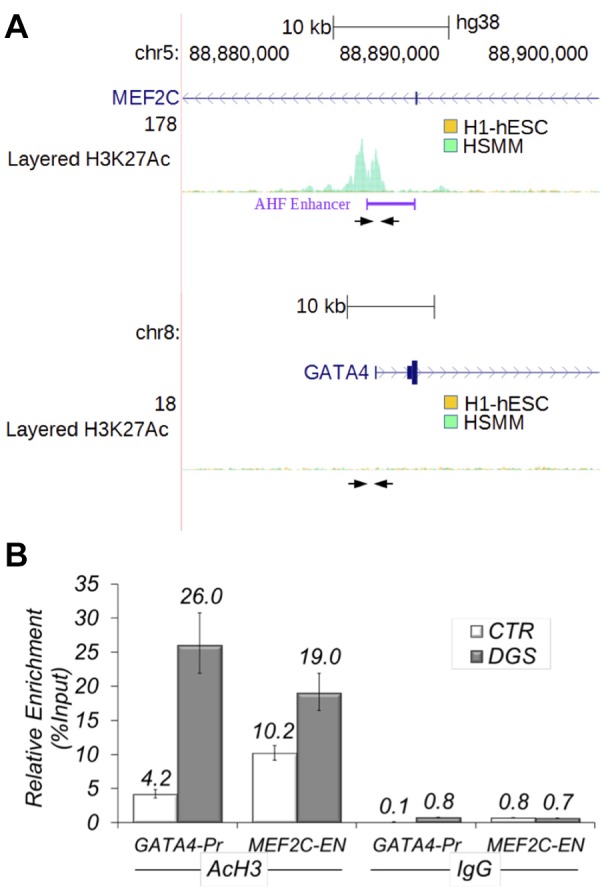
**Acetylation of histone 3 in *MEF2C* and *GATA4* loci is enhanced in differentiating 22q11.2DS hiPSCs.** (A,B) Localization of primer pairs (arrows) used for ChIP experiments with human cells on *MEF2C* and *GATA4* genes. ‘AHF Enhancer’ indicates a region of high similarity with the murine AHF enhancer. For reference, we report ENCODE ChIP-seq data for H3K27Ac for two cell types, undifferentiated human embryonic stem cells (H1-hESC) and human skeletal muscle myoblasts (HSMM). The *GATA4* promoter region is not acetylated in these two cell types. (C) ChIP assays with an anti-AcH3 antibody in differentiating control and 22q11.2DS hiPSCs, followed by quantitative real-time PCR.

Next, we performed q-ChIP assays on the AHF enhancer homologous region of the *MEF2C* gene and on the *GATA4* promoter. Results demonstrated that H3-Ac enrichment is significantly higher in 22q11.2DS cells compared with controls for both genes (Fig. 7).

## DISCUSSION

Overall, our data indicate that Tbx1 functions as a transcriptional repressor of the *Mef2c* AHF enhancer in mouse and human cells and, *in vivo*, in mouse embryos. In embryos, we noted that the response of the enhancer is *Tbx1* dosage dependent and is restricted to the splanchnic mesoderm, in a posterior-lateral domain, indicating context-dependent regulation. The AHF enhancer integrates the actions of several critical transcription factors (Dodou et al., 2004), e.g. Isl1, Nkx2-5, Gata4 and Tbx1. Interpreting the combinatorial code governing its regulation is of interest for the understanding of SHF development. We have previously shown that in cultured cells, Tbx1 inhibits Gata4-mediated activation of the AHF enhancer, but not Isl1-mediated activation (Pane et al., 2012). Interestingly, here we noted that the Mef2c-AHF-Cre expanded expression domain in mutant embryos is not stained with our Isl1 immunofluorescence, whereas most of the other Mef2c-AHF-Cre expression domains are Isl1 positive. In the future, it would be of interest to establish whether Isl1 represents a ‘protective factor’ against Tbx1-dependent suppression.

Ripply3 binds Tbx1 and inhibits its transcriptional activity in cell culture experiments (Okubo et al., 2011). In addition, and consistently, Ripply3 turns Tbx1 into a transcriptional repressor in *Xenopus* (Janesick et al., 2012). Ripply proteins can repress transcription through indirect interaction with HDAC1 (Brantjes et al., 2001; Jennings and Ish-Horowicz, 2008). However, in mouse, Ripply3 expression is restricted to the pharyngeal endoderm (Okubo et al., 2011), so it is unlikely to play a role in the observed expansion of the Mef2c-AHF-Cre domain in the splanchnic mesoderm. Groucho/TLE proteins can also mediate repression activity of Tbx15, Tbx18 and Tbx20 (Farin et al., 2007; Kaltenbrun et al., 2013), but Tbx1 lacks the Groucho interaction domain (Farin et al., 2007). Our data showed that Tbx1 does not interact directly with HDAC1 or HDAC2, suggesting the existence of molecular intermediates responsible for driving histone deacetylation. Alternatively, Tbx1 occupation of the enhancer might result in displacement of a transcription factor recruiting histone acetyl transferases.

Of particular interest is the embryonic region in which Tbx1 effects this function as it might be related to sorting anterior versus posterior heart field cells, and/or affect cardiomyocyte progenitor contribution to the OFT. Future studies should be directed to the identification of molecular intermediate(s) operating in the splanchnic mesoderm and affecting the transcriptional activity of Tbx1 in this region.

## MATERIALS AND METHODS

### Generation of hiPSCs

Neonatal skin fibroblasts from a 22q11.2DS/DiGeorge syndrome patient were obtained from the Coriell Institute (GM07215); control skin fibroblasts were obtained from an unrelated age- and sex-matched anonymous, healthy donor. Informed consent was reviewed by the Institutional Review Board of Baylor College of Medicine.

For hiPSC induction, retroviruses encoding the human *OCT3* (also known as *SLC22A3*), *OCT4* (also known as *POU5F1*), *SOX2*, *KLF4* and *c-MYC* (also known as *MYC*) factors were independently produced by transfecting HEK293T cells with pMXs vectors (Addgene plasmids 17217, 17218, 17219 and 17220) and the combination of moloney gag-pol plasmid pUMVC (Addgene plasmid 8449) and VSV envelope plasmid (Addgene plasmid 8454) in Dulbecco's modified eagle medium (DMEM) containing 10% fetal bovine serum (FBS) using Fugene HD (Roche, Penzberg, Germany). Viral supernatants were harvested after 48 h and 72 h, filtered through a 0.45-μm low-protein-binding cellulose acetate filter, concentrated by a spin column (Millipore, Billerica, MA, USA), and used directly to infect twice (24 h apart) 1.5×10^5^ human primary skin fibroblasts (PSFs) in the presence of 8 μg/ml polybrene. After 6 days, cells were seeded on mouse embryonic fibroblast (MEF) feeders at a density of 5×10^4^ cells/10-cm dish and cultured for 4 additional weeks in human ESC medium, consisting of DMEM/F12 supplemented with 20% knockout serum replacement (KSR, Invitrogen, Woltham, MA, USA), 2 mM L-glutamine, 0.1 mM nonessential amino acids, 0.1 mM β-mercaptoethanol, 50 U/ml penicillin, 50 mg/ml streptomycin and 10 ng/ml human b-FGF (R&D Systems, Minneapolis, WI, USA), before hiPSC colonies were manually picked. Karyotyping of the hiPSC lines was performed at the Cell Culture Facility of the Telethon Institute of Genetics and Medicine in Naples, Italy, using standard methods.

### Cardiac differentiation of hiPSCs

To generate hiPSC embryoid bodies (EBs), hiPSC colonies were dissociated into clumps using phosphate-buffered saline (PBS) containing 2.5 mg/ml trypsin (USB, Staufen, Germany), 1 mg/ml collagenase IV (Invitrogen), 20% KSR and 1 mM CaCl_2_ (10 min at 37°C) and maintained for 3 days in MEF-conditioned human ESC medium in low-attachment plates. For spontaneous differentiation, medium was then replaced with DMEM/F12 supplemented with 20% FBS, 2 mM L-glutamine, 0.1 mM nonessential amino acids, 0.1 mM β-mercaptoethanol, 50 U/ml penicillin and 50 μg/ml streptomycin, and EBs were analyzed at day 15 for expression of marker genes of the three different germ layers. To improve cardiac differentiation, ascorbic acid (50 μg/ml) was added to the medium, and EBs were plated at day 7 on gelatin-coated dishes for better detection of beating foci.

For induction of human cardiac progenitors, hiPSCs were seeded on MEF feeders and, 1 day later, treated for 4 days with 10 ng/ml human BMP2 (R&D Systems) and 1 μM SU5402 FGF receptor inhibitor (Calbiochem, Darmstadt, Germany) in RPMI 1640 medium supplemented with 2 mM L-glutamine and 2% B27 supplement without vitamin A (Invitrogen), as described previously (Leschik et al., 2008; Moretti et al., 2010). Conversion of human cardiac progenitors into myocytes and vascular cells (endothelial and smooth muscle cells) was induced by supplementing the culture medium with 50 μg/ml ascorbic acid and 10 ng/ml human VEGF (R&D Systems).

### Immunofluorescence and alkaline phosphatase activity assay

hiPSCs (undifferentiated or differentiated) were fixed in 3.7% (vol/vol) formaldehyde and subjected to immunostaining by using the following primary antibodies: anti-human NANOG (rabbit polyclonal, Abcam, Cambridge, UK; 1:500), Alexa Fluor 488-conjugated anti-human TRA1-81 (mouse monoclonal, BD Pharmingen, Franklin Lakes, NJ, USA; 1:20). Alexa Fluor 488-, 594- and 647-conjugated secondary antibodies specific to the appropriate species were used (Life Technologies, Carlsbad, CA, USA; 1:500). Nuclei were detected with 1 µg/ml Hoechst 33528. Direct alkaline phosphatase activity was analyzed using the NBT/BCIP alkaline phosphatase blue substrate (Roche), according to the manufacturer's guidelines. Microscopy was performed using the imaging systems (DMI6000-AF6000), filter cubes and software from Leica microsystems. Images were pseudo-colored using Adobe Photoshop.

### Mouse lines and immunofluorescence

Mef2c-AHF-Cre mice (Verzi et al., 2005) were crossed with *Tbx1*^+/lacZ^ mice (Lindsay et al., 2001) to obtain Mef2c-AHF-Cre;*Tbx1*^+/lacZ^ animals, which were then crossed with *Tbx1^flox^*^/flox^ (Xu et al., 2004) or *Tbx1*^+/lacZ^ mice. *Tbx1*^+/lacZ^ (also referred to as *Tbx1*^+/−^) mice were also crossed with wild-type mice to harvest embryos to be used for *Tbx1* gene expression by β-galactosidase assay. Animal studies were carried out under the auspices of the animal protocol 257/2015-PR (licensed to the A.B. laboratory) reviewed, according to Italian regulations, by the Italian Istituto Superiore di Sanità and approved by the Italian Ministero della Salute. Pregnant females (mostly 3-6 months old) at E9.5 were sacrificed using CO_2_ inhalation. The laboratory applies the ‘3Rs’ principles to minimize the use of animals and to limit or eliminate suffering.

Immunofluorescence on paraffin-embedded E9.5 embryo sections was performed using an anti-Cre antibody (69050-3, Novagen, Madison, WI, USA; 1:1000), and an anti-Isl1 antibody (39.4D5-s, Developmental Studies Hybridoma Bank, Iowa City, IA, USA; 1:50). β-galactosidase assays were performed on whole-mount embryos using salmon-gal (6-chloro-3-indolyl-beta-D-galactopyranoside, B21131, Alfa Aesar, Haverhill, MA, USA).

### qRT-PCR

Total mRNA was isolated from PSFs, hiPSCs, EBs and cardiac cells using the Stratagene Absolutely RNA kit and 1 µg was used to synthesize cDNA using the High-Capacity cDNA Reverse Transcription kit (Applied Biosystems, Foster City, CA, USA). Gene expression was quantified by qRT-PCR using 1 µl of the reverse transcription reaction and the Power SYBR Green PCR Master Mix (Applied Biosystems). Gene expression levels were normalized to *GAPDH*. A list of primers is provided in Table S1.

### Cell lines, plasmids and transfections

Mouse C2C12 myoblasts (CRL-1772, American Type Culture Collection) were cultured in DMEM supplemented with 10% FBS. Cultures were tested for mycoplasma infection and were negative. Authentication has been performed using differentiation marker analyses. For differentiation and transient transfection, cells were plated at a density of 1.5×10^5^ cells/well on a 35-mm tissue culture dish and incubated at 37°C in 5% CO_2_. After 24 h, the medium was replaced with a differentiation medium containing 2% horse serum (Hyclone, Marlborough, MA, USA). Transfection of 1 µg of Tbx1-3HA (Chen et al., 2012) was performed with X-tremeGENE (Roche) according to the manufacturer's instructions.

### ChIP

C2C12 cells and hiPSCs were crosslinked with 1% formaldehyde for 15 min at room temperature and glycine was added to stop the reaction to a final concentration of 0.125 M for 5 min. The cell pellet was suspended in 6× volumes of cell lysis buffer (10 mM HEPES, 60 mM KCl, 1 mM EDTA, 0.075% v/v NP40, 1 mM DTT and 1× protease inhibitors, adjusted to pH 7.6) in a 1.5 ml tube incubating on ice for 15 min. Isolated nuclei were suspended in Buffer B (LowCell ChIP Kit reagent) and chromatin was sheared into 200-500-bp long fragments using the Covaris S2 Sample Preparation System (duty cycle, 5%; cycles, 5; intensity, 3; temperature, 4°C; cycles per burst, 200; power mode, frequency sweeping; cycle time, 60 s; degassing mode, continuous). Soluble chromatin was incubated with 6 μg anti-acetyl-histone H3 (06-599, Millipore), anti-H3K27Ac (ab4729, Abcam) or normal rabbit/mouse IgG (sc-2027, Santa Cruz Biotechnology, Dallas, TX 75220, USA). Subsequent steps included extensive washes and reverse crosslinking following the LowCell ChIP Kit instructions. For quantitative ChIP, we performed real-time PCR of the immunoprecipitated DNA and inputs, using the FastStart Universal SYBR Green Master kit (Roche) and the 7900HT Fast Real-Time PCR System or StepOnePlus (Applied Biosystems) using primers specific for the AHF-enhancer region of *Mef2c* (Table S1).

*Tbx1* knockdown in C2C12 cells was obtained by transfection with nontargeted or *Tbx1*-targeted small interfering RNA (pool of s74767 and s74769, Life Technologies; 50 nmol/l) using Lipofectamine RNA iMAX (Life Technologies). Cells were crosslinked and processed for ChIP 48 h after transfection, as described above.

### Co-IP

C2C12 cells were transfected with aTbx1:3xHA expression vector (1 µg) and harvested after 24 h for protein extraction. Then, 100 μl protein-G Dynabeads (10004D, Life Technologies) were crosslinked to 20 μg anti-HA antibodies (11583816001, clone 12CA5, Roche) or control IgG (ab46540, Abcam). Nuclear extracts were incubated overnight at 4°C with crosslinked antibodies and extensively washed with PBS-0.02% Tween 20. Two consecutive elutions were performed with 0.1 M glycine (pH2.8) and immediately neutralized with Tris-HCl (pH 8.0). Samples were subjected to sodium dodecyl sulfate–polyacrylamide gel electrophoresis and proteins were transferred into polyvinylidene fluoride membrane for western blot analyses with anti-HDAC1 antibody (Ab7028, Abcam), anti-HDAC2 (sc-7899, Santa Cruz Biotechnology), anti-HA antibody (11583816001, clone 12CA5, Roche) and VeriBlot for IP secondary antibody (HRP) (ab131366, Abcam; 1:200).

### Statistical analysis

All data were expressed as means±s.e.m. (or s.d., as indicated in the figure legends) from independent experiments. Differences between groups were examined for statistical analysis using two-tailed Student's *t*-test. *P*<0.05 was considered significant.

## Supplementary Material

Supplementary information
