# Supplementary material for: Tbx1 represses Mef2c gene expression and is correlated with histone 3 deacetylation of the anterior heart field enhancer
Source: Dis Model Mech. 2018 Aug 30;11(9):dmm029967. doi: 10.1242/dmm.029967 (PMC6176997; doi:10.1242/dmm.029967)
Supplement: Supplementary information [file dmm-11-029967-s1.pdf]

SUPPLEMENTARY TABLE 1

Table S1: Sequence of oligonucleotide primers used in this study.

Name	Use	Sequence 5'-3'	
hOCT3/4Tg	qRT-PCR	For	GCTCTCCCATGCATTCAAAC
		Rev	TTATCGTCGACCACTGTGCTGCTG
hSOX2Tg	qRT-PCR	For	GGGAAATGGGAGGGGTGCAAAAGAGG
		Rev	TTGCGTGAGTGTGGATGGGATTGGTG
hKLF4Tg	qRT-PCR	For	CCACCTCGCCTTACACATGA
		Rev	CCCTTTTTCTGGAGACTAAATAAA
hc-MYC endo	qRT-PCR	For	AGAAATGTCCTGAGCAATCACC
		Rev	AAGGTTGTGAGGTTGCATTTGA
hKLF4 endo	qRT-PCR	For	ATAGCCTAAATGATGGTGCTTGG
		Rev	AACTTTGGCTTCCTTGTTTGG
hOCT3/4 endo	qRT-PCR	For	GACAGGGGGAGGGGAGGAGCTAGG
		Rev	CTTCCCTCCAACCAGTTGCCCCAAAC
hSOX2 endo	qRT-PCR	For	GGGAAATGGGAGGGGTGCAAAAGAGG
		Rev	TTGCGTGAGTGTGGATGGGATTGGTG
hNANOG	qRT-PCR	For	TGCAAGAACTCTCCAACATCCT
		Rev	ATTGCTATTCTTCGGCCAGTT
hREX1	qRT-PCR	For	ACCAGCACACTAGGCAAACC
		Rev	TTCTGTTCACACAGGCTCCA
hTDGF1	qRT-PCR	For	CCCAAGAAGTGTTCCCTGTG
		Rev	ACGTGCAGACGGTGGTAGTT
hPDX1	qRT-PCR	For	AAGCTCACGCGTGGAAAG
		Rev	GGCCGTGAGATGTACTTGTTG
hSOX7	qRT-PCR	For	TGAACGCCTTCATGGTTTG
		Rev	AGCGCCTTCCACGACTTT
hAFP	qRT-PCR	For	GTGCCAAGCTCAGGGTGTAG
		Rev	CAGCCTCAAGTTGTTCCTCTG
hCD31	qRT-PCR	For	ATGCCGTGGAAAGCAGATAC
		Rev	CTGTTCTTCTCGGAACATGGA
hDES	qRT-PCR	For	GTGAAGATGGCCCTGGATGT
		Rev	TGGTTTCTCGGAAGTTGAGG
hACTA2	qRT-PCR	For	GTGATCACCATCGGAAATGAA

		Rev	TCATGATGCTGTTGTAGGTGGT
hSCL	qRT-PCR	For	CCAACAATCGAGTGAAGAGGA
		Rev	CCGGCTGTTGGTGAAGATAC
hMYL2	qRT-PCR	For	TACGTTCGGGAAATGCTGAC
		Rev	TTCTCCGTGGGTGATGATG
hCDH5	qRT-PCR	For	GAGCATCCAGGCAGTGGTAG
		Rev	CAGGAAGATGAGCAGGGTGA
hKRT14	qRT-PCR	For	CACCTCTCCTCCTCCCAGTT
		Rev	ATGACCTTGGTGCGGATTT
hNCAM1	qRT-PCR	For	CAGATGGGAGAGGATGGAAA
		Rev	CAGACGGGAGCCTGATCTCT
hTH	qRT-PCR	For	TGTACTGGTTCACGGTGGAGT
		Rev	TCTCAGGCTCCTCAGACAGG
hGABRR2	qRT-PCR	For	CTGTGCCTGCCAGAGTTTCA
		Rev	ACGGCCTTGACGTAGGAGA
hGAPDH	qRT-PCR	For	TCCTCTGACTTCAACAGCGA
		Rev	GGGTCTTACTCCTTGGAGGC
hTBX1	qRT-PCR	For	CACATGGAGTTGTCGTGTTTCC
		Rev	TTC AAG AGC ACG TCC AGC AA
hISL1	qRT-PCR	For	AAAGTTACCAGCCACCTTGGA
		Rev	ATTAGAGCCCGGTCCTCCTT
hNKX2.5	qRT-PCR	For	CAAGTGTGCGTCTGCCTTT
		Rev	TTGTCCGCCTCTGTCTTCTC
hGATA4	qRT-PCR	For	GGCCTGTCATCTCACTACGG
		Rev	ATGGCCAGACATCGCACT
hMEF2C	qRT-PCR	For	ATCTGCCCTCAGTCAGTTGG
		Rev	AGAAGGCAGGGAGAGATTTGA
hCTNT	qRT-PCR	For	AGCATCTATAACTTGGAGGCAGAG
		Rev	TGGAGACTTTCTGGTTATCGTTG
mMef2c-ChIP	ChIP	For	TGAGGAGGGAGCTGCAGTAT
		Rev	CCGTTTCTCTATCCCAACCA
hMef2c-ChIP	ChIP	For	TGTGACTTTTCCCCCTTTTG
		Rev	TCCTGGACCGTGGTTTTTAC
mGata4ChIP	ChIP	For	AGAGCAGCAAACCGCAAG
		Rev	AGGACTCTTCCCAAAGCTC
hGataChIP	ChIP	For	GGTGTCTCCTGAACCTCCAA

		Rev	TGGGCTCAACTCTCGATCTT
mINT-XIV-1	ChIP	For	TTCTTGTCCACAGCCCTCTT
		Rev	TGGTGGAAGAGGAGACATCC
hORF-Free-CT	ChIP	For	AGTGATGGGCCAAACTGAAG
		Rev	AGGGGCAGAGAGATGACAGA

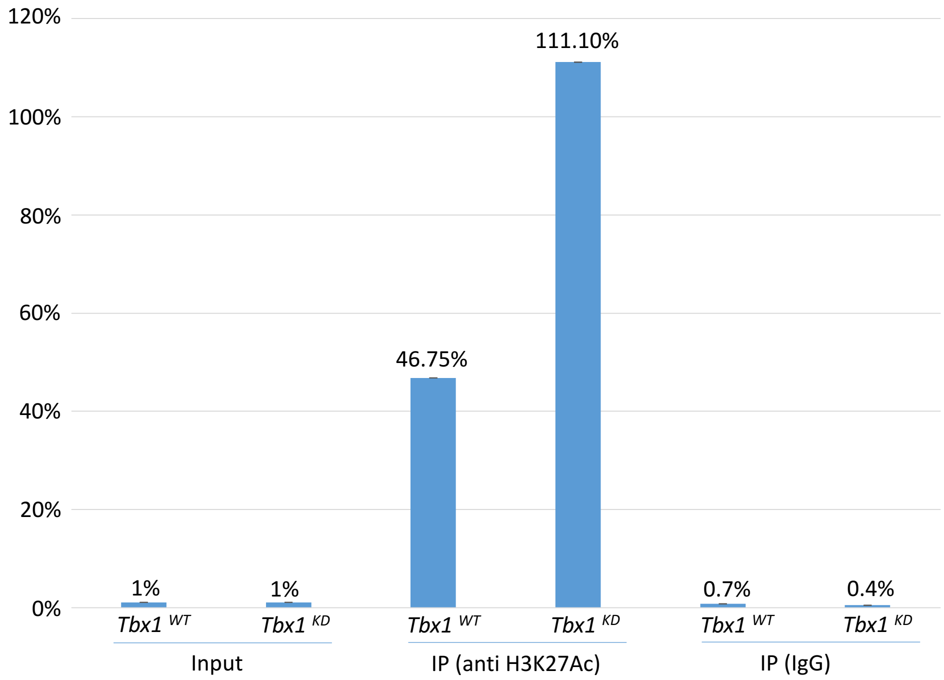

Figure S1

Histogram showing the results of Q-ChIP analyses using anti H3K27Ac antibodies on C2C12 cells treated with non-targeting siRNA (Tbx1WT) or with Tbx1-targeted siRNA (Tbx1KD) on the same locus as the one tested in Fig. 2. Enrichment is shown as percentage of input. Results are the mean of two biological replicates (error bars indicate s.e.m.).

Disease Models & Mechanisms • Supplementary information

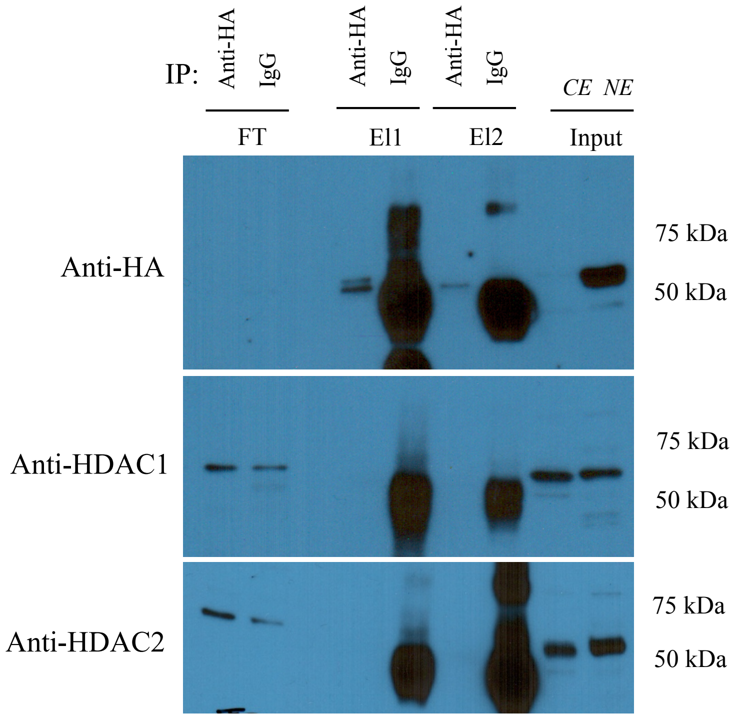

Figure S2

Western blot analysis of representative immunoprecipitation experiment using protein extracts from Tbx1:3xHA-transfected C2C12 cells. Immunoprecipitation of nuclear extracts was performed using anti-HA antibodies or IgG (control). Top panel: control WB using anti-HA antibodies. Lower panels: WB with anti-HDAC1 and anti-HDAC2 antibodies. Immunoreactivity is found in the FT and input samples but not in the immunoprecipitated samples (El1 and El2). The anti-HDAC2 experiment shown was performed on the same immunoprecipitated material as anti-HDAC1 and anti-HA, but run on a different gel.

The experiment has been repeated 5 times. FT: flow-through; El1: Elution 1; El2: Elution 2; CE: cytoplasm extract; NE: nuclear extract.

Disease Models & Mechanisms • Supplementary information

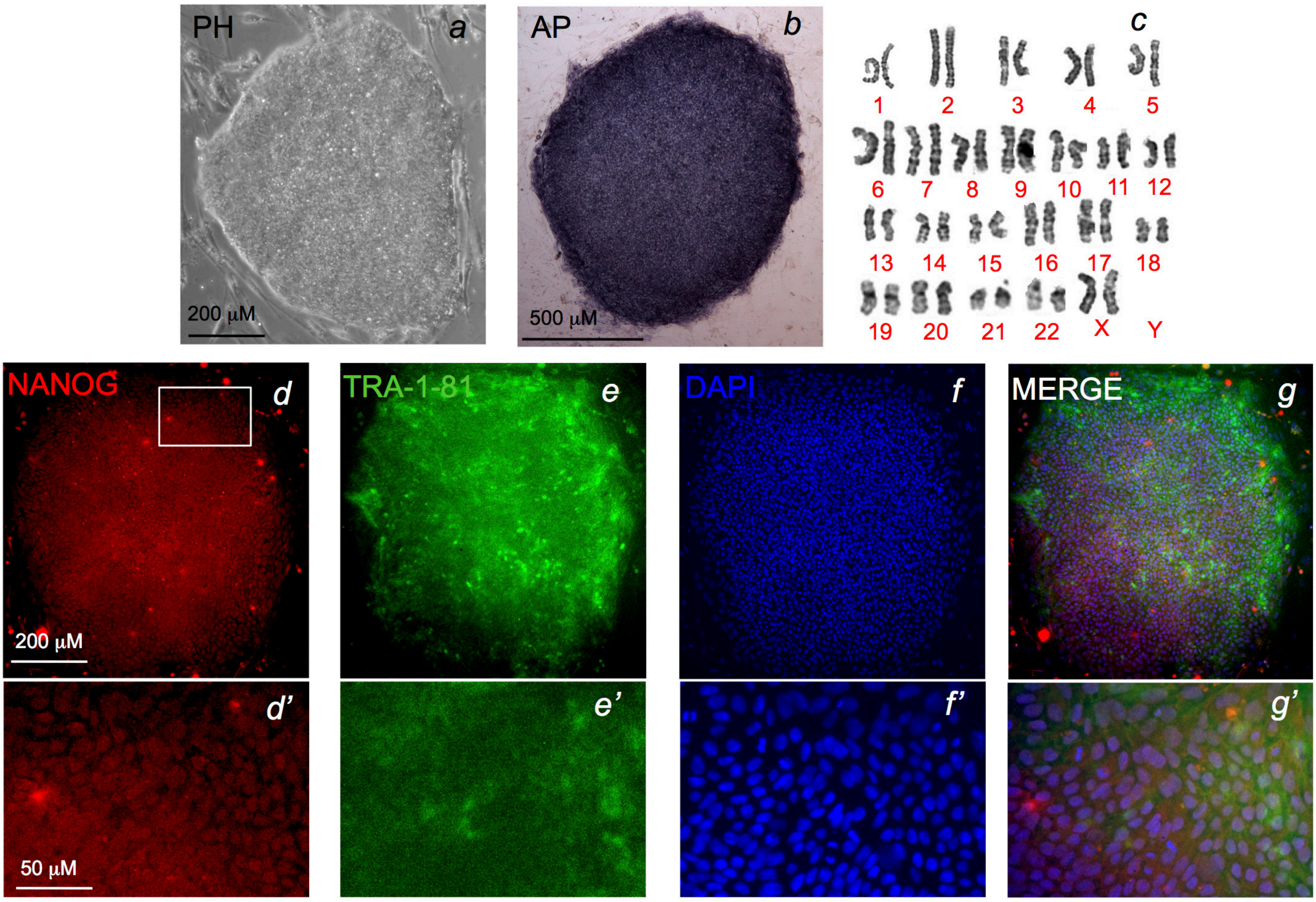

Figure S3
Generation and characterization o 22q11.2DS patient-derived iPSCs.
Images of colonies from a representative 22q11.2DS iPSC clone in bright field (a) and after staining for alkaline phosphatase (AP) activity (b). Scale bar, 500 and 200 µm respectively. c, Karyotyping of a representative 22q11.2DS iPSC clone. d-f', Immunofluorescence analysis of pluripotency markers NANOG (d,d') and TRA1-81 (e, e') in a representative iPSC clone. DNA is stained with DAPI (f,f'). Scale bar, 200 µm. d', e' and f' are magnification of d, e and f respectively. Scale bar, 50 µm.

Disease Models & Mechanisms • Supplementary information

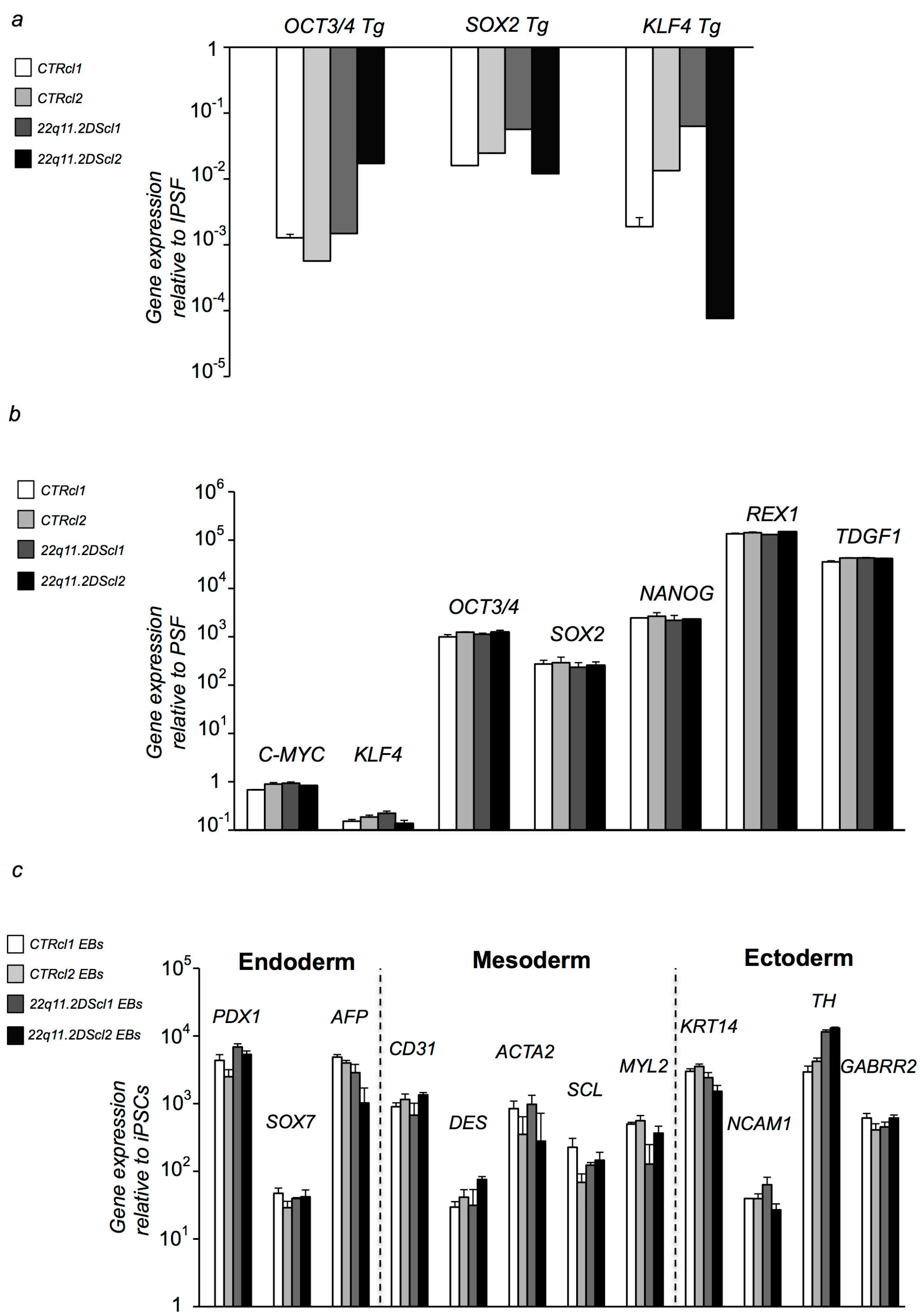

Figure S4

Assessment of pluripotency in control and 22q11.2DS iPSCs a, Quantitative RT-PCR (qRT-PCR) for expression of retroviral transgenes in two iPSC clones from a control individual (CTRcl1 and CTRcl2) and two from a 22q11.2DS patient (22q11.2DScl1 and 22q11.2DScl2). Expression values are relative to the corresponding primary skin fibroblasts (PSF) infected with the four retroviruses OCT3/4, SOX2 and KLF4 (Infected prmary skin fibroblasts, IPSF), normalized to GAPDH, and presented as mean ± s.e.m., n=3. b, qRT-PCR analysis of endogenous genes associated with pluripotency (c-MYC, KLF4, OCT3/4, SOX2, NANOG, REX1, and TDGF1) in the two control (CTRcl1 and CTRcl2)and two 22q11.2DS iPSC clones (22q11.2DScl1 and 22q11.2DScl2). Expression values are relative to corresponding PSF, normalized to GAPDH, and presented as mean ± s.e.m., n=3. c, qRT-PCR analysis of markers of the three germ layers, endoderm (PDX1, SOX7, and AFP), mesoderm (CD31, DES, ACTA2, SCL, MYL2, and CDH5), and ectoderm (KRT14, NCAM1, TH, and GABRR2) in embryoid bodies (EBs) at day 21 of differentiation from the two control (CTRcl1 and CTRcl2 EBs) and two 22q11.2DS iPSC clones (22q11.2DScl1 and 22q11.2DScl2 EBs). Expression values are relative to the corresponding undifferentiated iPSC clones, normalized to GAPDH, and presented as mean ± s.e.m., n=3.

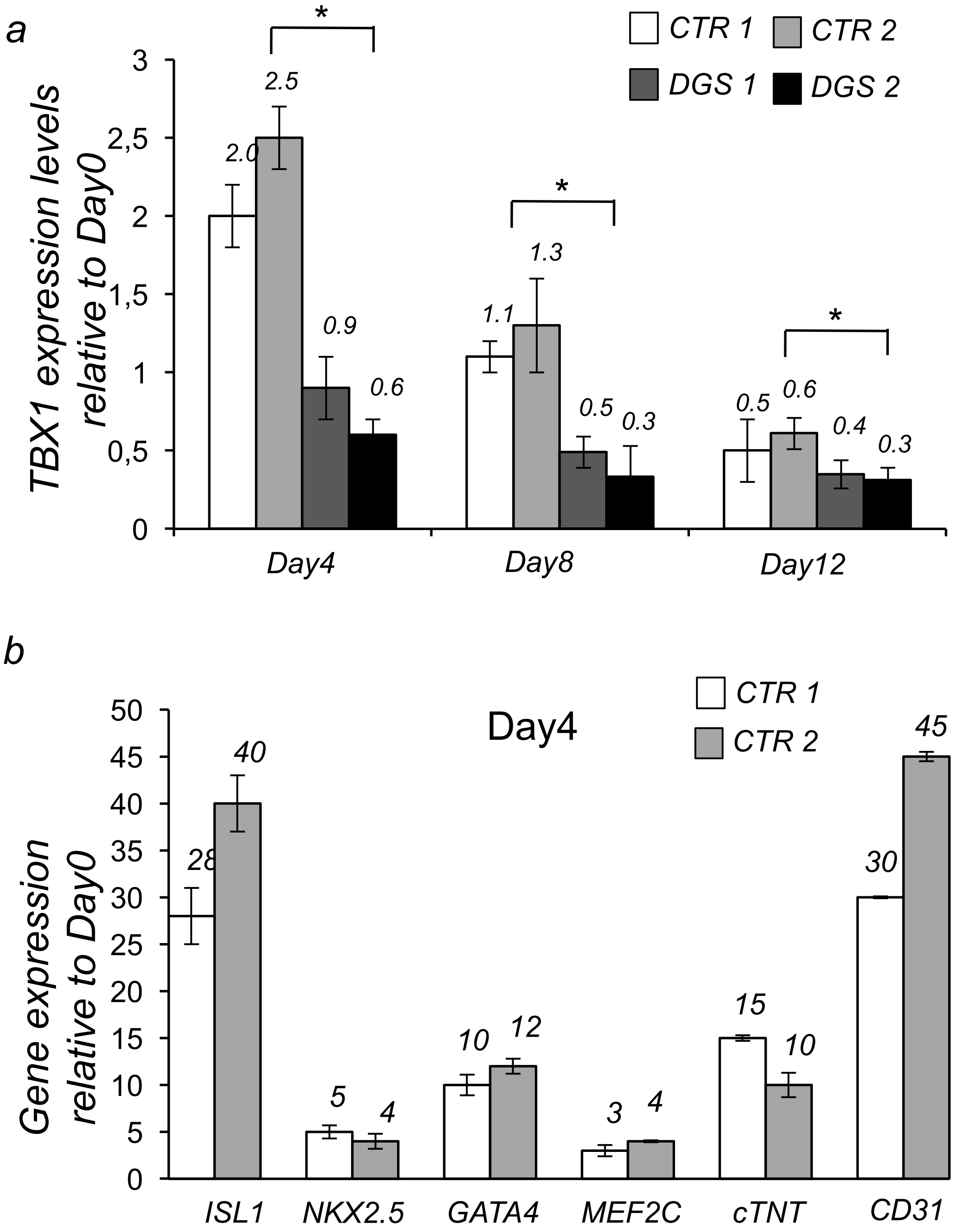

Figure S5

Analysis of TBX1 expression in control and 22q11.2DS iPSC-derived cardiac cells. a, Quantitative RT-PCR (qRT-PCR) -based TBX1 expression profile in two iPSC clones from a control individual and two from a 22q11.2DS patient during cardiac differentiation.

b, qRT-PCR-based comparison of expression of the genes indicated in the two control iPSC clones after 4 days BMP2/FGFR-inhibitor treatment (Day4). n=3,*P<0.05 Vs. Day 0.
